# Editorial: Stress and addictive disorders

**DOI:** 10.3389/fpsyt.2023.1307732

**Published:** 2023-10-31

**Authors:** Andrew Chih Wei Huang, Chih-Yuan Ko, Anna Kozłowska, Bai-Chuang Shyu

**Affiliations:** ^1^Department of Psychology, Fo Guang University, Yilan, Taiwan; ^2^Department of Clinical Nutrition, The Second Affiliated Hospital of Fujian Medical University, Quanzhou, China; ^3^Department of Human Physiology and Pathology, School Medicine, Collegium Medicum, University of Warmia and Mazury in Olsztyn, Olsztyn, Poland; ^4^Institute of Biomedical Sciences, Academia Sinica, Taipei, Taiwan

**Keywords:** stress, addictive behavior, drug addiction, psychological intervention, pharmacological intervention

Stress events include chronic stress, acute stress, traumatic stress, natural disasters (e.g., earthquakes), sex abuse, rape, wars, and severe diseases that cause death (e.g., cancer and COVID-19) (1–4). The various types of stress events can induce impulsive and compulsive behaviors and cause vulnerability to addictive behaviors. Accordingly, many reports indicated that stress can potentially enhance the development of addictive and relapse behaviors (5, 6). In the animal model, growing body evidence showed that experiencing stress events can increase drug self-administration and drug-seeking behaviors (7–9). In these cases, chronic or acute stress alters the mesolimbic dopamine, glutamate, noradrenaline, and GABA systems; moreover, it increases the corticotropin-releasing factor of the hypothalamus-pituitary-adrenal axis and the autonomic hyperactivity (3, 10–13). Recently, the kappa receptor and endogenous ligand dynorphin were found to contribute to the occurrence of comorbidity for substance abuse with chronic stress, post-traumatic stress disorder (PTSD), and traumatic stress, indicating the opiate system is also involved in the stress-induced anxiety and addictive behaviors (14).

In particular, the reward and reinforcement pathway of the ventral tegmental area to the nucleus accumbens was sensitized by chronic stress treatments. By way of the neural sensitization in the reward system, stress contributes to the vulnerability of addictive behaviors and enhances its prevalence. Therefore, stress is highly associated with the occurrence of addictive behaviors.

How to prevent and terminate addictive behaviors remains a big issue. Until now, some pharmacotherapy and psychotherapy have been developed to ameliorate addiction, effectively (15, 16). For example, some studies have reported that psychotherapies might be effective in reducing addictive behaviors, including cognitive-behavior therapy (17), motivational enhancement therapy, behavioral enhancement therapy, psychological addiction elimination technology, mindfulness-based relapse prevention, unconditioned stimulus memory retrieval-extinction paradigm, program implantation technology under deep hypnosis, aversion therapy, individual therapy, and group therapy (15). Moreover, psychotherapy was applied to prevent and treat Internet addiction (18). Obviously, psychotherapy is an effective treatment for reduction of addictive behaviors.

Alternatively, pharmacological treatments were alternative considerations for ameliorating addictive behaviors (16, 19). For example, opiate antagonists (e.g., naloxone) (20) and agonists (e.g., methadone) (21) can reverse or lessen opiate addiction. Previous studies have reported that the approval of the USA Food and Drug Administration for medications in treatments of nicotine, alcohol, and opiate abuse by ways of affecting dopamine, GABA, serotonin, and glutamate systems in the brain (16, 19). Downregulation of the brain's dopamine levels can relieve the incentive property and reinforcement or reward of the addictive stimulus, resulting in the amelioration of drug and food addiction (22). In conclusion, psychotherapy and pharmacotherapy are essential strategies for treating drug and non-drug addictive behaviors.

In our Research Topic, some studies demonstrated that numerous stress events facilitated addictive behaviors. For example, Obuobi-Donkor et al. found that people who experienced multiple natural disasters had a high vulnerability and prevalence risk of cannabis abuse and anxiety and depression symptoms. Nin et al. showed that lower social distancing increased abused drugs during the pandemic; however, anxiety and depression were associated with higher drug use for sociodemographics (such as men, lower income, and less education). Thus, the COVID-19 pandemic-induced stress changes the use of abused drugs and the mental state. Zhang et al. revealed that perceived stress was associated with lower self-control due to a high risk of mobile phone addiction; however, the security factor moderated the relationship between perceived stress and self-control.

On the other hand, novel interventions were developed to reduce stress-induced PTSD symptoms and addictive behaviors. For example, Yu et al. used the optogenetics approach to demonstrate that the different subareas of the medial prefrontal cortex moderated alternations of the stimulus valence from reward to aversion or neutral states, and the findings can develop novel treatments for drug addiction. Lee et al. employed the PTSD animal study, which showed that the blockade of hypocretin signals in the basolateral amygdala reduced the PTSD-like behaviors induced by a novel stress protocol in mice. Their data indicated the hypocretin system plays an essential role in modulating PTSD symptoms, indicating that hypocretin can be developed as a new treatment for PTSD. Altogether, all collected research as above has shown their findings and conclusions in Table 1.

**Table 1:** Summary for the topic research related to the issues of stress and behavioral addiction and its interventions for addiction.

	**Obuobi-Donkor et al.**	**Nin et al.**	**Zhang et al.**	**Yu et al.**	**Lee et al.**
Stress and addictive behavior	Multiple natural disaster-induced stress affects cannabis abuse	COVID-19/Social distancing and drug abuse	Perceived stress and self-control	N/A	N/A
Interventions	N/A	N/A	N/A	Optogenetic approach test the functions of the mPFC to change stimulus valence and developing new interventions.	Role of the hypocretin system in stress and developments in novel treatments.

N/A, Not applicable.

In summary, the topic research manipulates numerous stress events and examines how stress alters addictive behaviors. Moreover, novel interventions were developed to alleviate stress-induced PTSD symptoms and addictive behaviors. The present findings can help us understand the brain mechanisms and provide some contributions and implications in clinical aspects.

## Author contributions

AH: Conceptualization, Writing—original draft, Writing—review & editing. C-YK: Writing—review & editing. AK: Writing—review & editing. B-CS: Writing—review & editing.
